# Public Health Center Strategies to Develop Infection Control Practices for Hospital Sinks in Kawaguchi City

**DOI:** 10.7759/cureus.91088

**Published:** 2025-08-27

**Authors:** Ayako Nakayama, Ichiro Yamaguchi, Koji Okamoto, Shigefumi Maesaki

**Affiliations:** 1 Administration Department, Kawaguchi Public Health Center, Kawaguchi, JPN; 2 Environmental Health Department, National Institute of Public Health, Saitama, JPN; 3 Infectious Disease and Infection Control Department, Saitama Medical University, Saitama, JPN

**Keywords:** effects by training sessions, hospitals, infection control practices, multidrug-resistant organisms, sinks, the public health center, training sessions

## Abstract

Introduction

The Kawaguchi City Public Health Center (PHC) conducted training sessions (TSs) focusing on infection control (IC) practices for multidrug-resistant organisms (MDROs) at healthcare facilities (HCFs) in its jurisdiction in June 2023. We planned to examine whether the TS programs by the PHC have had any effect on the development of IC practices for hospital sinks, to support the hospitals in their development of IC practices to prevent MDRO infections. In this study, we aim to identify effective PHC strategies for developing hospital sink IC practices using post-TS survey results.

Methods

In June 2023, we completed a TS for 30 HCFs, offered information on IC practices, and then sent out a questionnaire. We examined both current IC practices and practices that the hospitals intended to conduct for sinks and identified the first learned information from the TSs organized by the PHC.

Results

Twenty-four HCFs responded to the survey, a response rate of 80.0%. Current IC practices for hospital sinks included refraining from disposing substances like medications and liquid food (refrain from disposing) by 16 hospitals (66.7%), dedicating sinks for handwashing (dedicate sinks) by 14 hospitals (58.3%), installing splash guards by two hospitals (8.3%), and keeping supplies off surfaces within 1 m of the sinks (keep supplies off) by two hospitals (8.3%). IC practices that hospitals planned to conduct for sinks included refraining from disposing by two hospitals (8.3%), dedicating sinks by two hospitals (8.3%), installing splash guards by six hospitals (25.0%), and keeping supplies off by seven hospitals (29.2%). Components of the first learned information included the effects of cleaning bundles by 10 hospitals (41.7%), knowledge sharing organized by the PHC for four hospitals (16.7%) and the effect of TSs on IC, such as a decrease in MDROs associated with TSs (the effect of TSs) by three hospitals (12.5%).The first piece of learned information “the effect of TSs (evidence that TSs reduce MDRO infections)” was significantly associated with the intention of dedicating sinks (p = 0.008). Other planned IC practices for sinks such as refraining from disposing substances, installing splash guards, and keeping supplies off were not associated with the TS programs.

Conclusions

Planned IC practices such as dedicating sinks for handwashing were linked to providing information on the effects of the TSs. We suggest that the PHC should develop TSs, including information about the effects of TSs, for IC practices that cannot be strengthened without staff support.

## Introduction

The World Health Organization (WHO)’s World Health Assembly in May 2015 adopted a worldwide action plan on antimicrobial resistance (AMR) [1], which represents the consensus that AMR could pose a serious threat to human health all over the world if no action is taken against multidrug-resistant organisms (MDROs). In Japan, the government created the National Action Plan (NAP) on AMR to run from 2016 to 2020. Moreover, it renewed the NAP on AMR from 2023 to 2027 to combat AMR. As a result, Japan has had a lower incidence of carbapenemase-producing *Enterobacteriaceae *(CPE), a type of carbapenem-resistant *Enterobacteriaceae *(CRE), at 0.17 cases per 100,000 population in 2022, which is quite low [2-3]. Nevertheless, CRE was identified in 45.2% of medical facilities in 2022, and the identified CRE cases numbered over 9,000 per year, according to the Japan Nosocomial Infections Surveillance (JANIS), as reported by 25% of Japan’s hospitals [4]. There is a concern that the number of identified CRE cases might increase in the future.

The WHO stated that outbreaks of carbapenem-resistant organisms among patients appeared to be more commonly associated with environmental contamination involving wastewater systems, such as sinks [5]. Additionally, outbreaks of MDRO infections were associated with contaminated sinks [6]. Nevertheless, the WHO does not provide guidelines on infection control (IC) for hospital handwashing sinks [5]. In the US, there is a guideline on IC for handwashing sinks that recommends the following activities: dedicate sinks to handwashing, refrain from disposing substances like medications and liquid food in handwashing sinks, do not keep medications or patient care supplies on countertops within 1 m of sinks, and install splash guards [7]. Moreover, CRE and CPE plasmid genes and bacterial strains from the patients have been linked to sinks in patients’ rooms [8]. A recent report has indicated that CPE can be transmitted from sinks to patients [9]. Hospitals have attempted to reduce gram-negative bacilli acquisition by removing sinks and applying filters on taps to address sink-related gram-negative bacilli outbreaks [10]. The IC practices for sinks recommended by the US guidelines have been implemented to terminate gram-negative bacilli outbreaks [10]. To our knowledge, there are no survey reports on IC practices for hospital sinks. In Kawaguchi City, which has a population of roughly 600,000, no study on IC for hospital sinks has ever been conducted before the survey and study in 2023 [11]. This situation has led us to conduct a survey on IC practices for sinks. We report that the public health center (PHC) supported the hospitals’ development of IC practices for sinks by conducting a survey on IC for sinks and examining the results.

In a study on IC by the hospital in Kawaguchi City conducted in 2022 and 2023, we indicated that providing information on numerical effects and quantities may help hospitals develop IC programs such as hand hygiene compliance programs (HHCPs) [11-12]. Moreover, we indicated that appropriate support from hospital leadership might contribute to the development of IC programs [11]. Nevertheless, training sessions (TSs) or strategies by the PHC that might develop IC practices for sinks have never been examined [11-12]. The purpose of this study is to clarify whether the strategy implemented by the PHC has been effective in developing IC practices for sinks by examining the results of a survey conducted after the TSs. In this study, we aim to assess the impact of TSs on hospitals’ intentions to implement IC practices for sinks, such as dedicating sinks to handwashing, installing splash guards, and avoiding nutrient disposal in sinks. Therefore, in this study, we attempted to determine which TS programs by the PHC had any effect on the development of IC practices for hospital sinks by using the survey results to analyze the relationship between the IC practices that the hospitals intended to conduct for sinks (IC practices to be conducted for sinks) and the first learned information from the TSs organized by the PHC. Furthermore, we carried out correlation analysis to determine the relationships between IC practices to be conducted for sinks and useful information or activities for developing IC.

## Materials and methods

In June 2023, there were 20 hospitals and 11 affiliated clinics (AFs) with beds in Kawaguchi. The director of the PHC emailed all the hospital administrators in the area. They informed them about the center’s training course and asked them to respond if they were interested in participating. Twenty hospitals and 10 AFs with beds in Kawaguchi applied for TSs [11]. In June 2023, the Kawaguchi City PHC conducted a TS to develop IC for MDROs for these 20 hospitals and 10 AFs with beds via a web conference system facilitated by a public health physician (Tables 1-2, Appendix) [11]. TS programs included enhancing HHCPs and environmental cleaning (EC) and IC for hospital sinks, and TSs (Table 1 and Table 2, Appendix). TS lasted 60 minutes. Prior to the session, there was an informational meeting on regular medical facility audits based on the Medical Care Act. Each administrator received an ID and password for the web conferencing system, allowing each participant to access the training individually.

**Table 1:** Outline of training session programs on IC and prevention of MDROs by Kawaguchi City Public Health Center IC: infection control; MDROs: multidrug-resistant organisms; EC: environmental cleaning

Training session programs
Enhancing hand hygiene compliance programs
Enhancing EC
Enhancing IC for hospital sinks
Enhancing training sessions
Knowledge sharing organized by the public health center
Evidence on preparing outbreaks
Necessity and evidence for implementing measures to control MDROs throughout the jurisdiction

**Table 2:** Training session programs on IC and prevention of MDROs by Kawaguchi City Public Health Center IC: infection control; MDROs: multidrug-resistant organisms; EC: environmental cleaning; ICU: intensive care unit; CPE: carbapenemase-producing *Enterobacteriaceae*; MRSA: methicillin-resistant *Staphylococcus aureus*; MDRA: multidrug-resistant *Acinetobacter*

Description	Training session programs
Hand hygiene compliance programs	Performance feedback may increase adherence to hand hygiene practices and reduce infection and colonization rates
	A ward-level hand hygiene compliance program can be successful in improving hand hygiene rates
	Feedback on the mean compliance rate for the past seven days should be provided
	Individual compliance rates were publicly posted in designated areas
	Simulation training sessions were effective for hand hygiene compliance programs
	Each ward should be responsible for setting its own goal for the hand hygiene rate
	Hand hygiene champions, doctors, and nurses should lead hand hygiene compliance programs
	Targets of 10–20% hand hygiene improvement were set
Evidence on enhanced EC	Admission to an ICU room previously occupied by a patient with MDROs was a risk factor for acquisition of the bacteria by subsequent room occupants
	Despite implementing enhanced IC, including contact precautions, the transmission of CPE through indirect ward and hospital contact did not decrease
	Genomic sequencing of isolates from patients revealed links to previous CPE outbreak strains from two years prior
	Implementing the cleaning bundle resulted in a decrease in MDROs and cost savings
Evidence for dust removal and EC that hospitals should conduct	We recommend that the hospitals remove dust in relevant areas by mopping, in reference to IC policies by university hospitals
	During the outbreak, the single MRSA strain was widespread in the dust in the ward environment
	The MDRA outbreak strains were isolated from the dust of respiratory ventilators and continuous veno-venous hemofiltration
Evidence for cleaning sinks and drains and EC that hospitals should conduct	Do not keep medications or patient care supplies on surfaces that are within 1m of sinks
	A splash barrier should be used
	The sink has a basin depth of at least 240 mm
	Covers should be put on sink drains
	CPE plasmids from the patient were linked to those from two sinks in the patient’s room
Evidence for monitoring of and feedback about EC	The possibility of transmission through inanimate objects could not be ruled out in the MDRO outbreak
	Monitoring of and feedback from EC is recommended
	Enhanced EC practices, including monitoring and feedback, and hiring new personnel can reduce MDROs infection rates
	EC monitoring was conducted using a marking gel that fluoresced when exposed to ultraviolet light, and feedback was provided to the EC staff and the manager in the previous study in which the health-associated infection decreased
Knowledge sharing organized by the public health center	The hospitals discussed the difficulties and barriers to developing IC in knowledge sharing organized by the public health center
Evidence on holding training sessions	A higher percentage of staff with IC training was associated with a decrease in MRSA infections
	IC training within the last three years was associated with higher knowledge scores regarding MDROs

After the public health doctor gave their presentation, they took questions from the participants. The TS participants were medical doctors, pharmacists, nurses, and hospital clinical laboratory technicians [11]. We e-mailed a questionnaire (Table 3) to the directors of 20 hospitals and 10 AFs with beds and requested permission to participate in the questionnaire and study after their staff finished the TSs. We indicated to the hospitals (a) that the questionnaire aimed to collect basic information for examining IC in facilities under the jurisdiction of Kawaguchi PHC and for determining which IC should be conducted, (b) that the aggregated questionnaire results would be returned as feedback to the hospitals after they were processed to protect the names of the hospitals, (c) that the results may be published on the city’s website or in academic journals, and (d) that we would edit them to prevent identification of the names of the hospitals and those who had filled out the responses before publication. Response to the questionnaire was considered as consent to participate in this study.

**Table 3:** Questionnaire on IC and prevention of MDROs by Kawaguchi City Public Health Center IC: infection control; MDROs: multidrug-resistant organisms; ICU: intensive care unit; CPE: carbapenemase-producing *Enterobacteriaceae*; MRSA: methicillin-resistant *Staphylococcus aureus*; MDRA: multidrug-resistant *Acinetobacter* ; EC: environmental cleaning

Description	Answer options
Regarding IC for handwashing sinks in your facility, please circle only those that apply to you regarding the IC currently implemented and those you intend to implement	Dedicating sinks for handwashing
	Install splash guards
	Install sink with side guards
	Install wide-neck faucets for staff use
	The faucets are wall-mounted to prevent contamination of the surrounding area
	Install washbasins more than 24 cm deep
	Keeping supplies off surfaces within 1 m of sinks
	Install sink without an overflow hole
	Install automatic faucets
	Install faucets that prevent water from flowing directly into the drain
	The edge of the sink is sloped to avoid placing items on it
	Provide feedback on the evaluation of environmental cleaning
	Refraining from disposing substances like medications and liquid food
Please circle all that apply regarding the information you learned for the first time in the training session.	Specific feedback effective for hand hygiene compliance programs
	Simulation training sessions were effective for hand hygiene compliance programs
	Admission to an ICU room previously occupied by a patient with MDROs was a risk factor for acquisition by subsequent room occupants
	Despite implementing enhanced IC including contact precautions, the transmission of CPE through indirect ward and hospital contact did not decrease
	Sequencing of CPE isolates from patients revealed links to previous outbreak strains
	Genomic analysis revealed that two patients who had been admitted to the facilities organized by the same healthcare systems and were not epidemiologically linked had almost identical strains and the same plasmid genes
	MRSA strain was widespread in the dust in the ward environment in the outbreak
	The MDRA strain detected in dust inside the ventilator was the same as that found in the patient
	CPE identified from the patient was almost identical to the plasmid gene of the bacteria identified in the sink of the patient's room. CPE identified from the patient was almost identical to the plasmid gene of the bacteria identified in the sink of the patient's room
	Implementing the cleaning bundle resulted in a decrease in MDROs and cost savings
	Enhanced EC practices were effective for IC
	Knowledge sharing organized by the public health center
	The training sessions were effective for IC
	Enhancing contact precautions without implementing a dedicated room in the ICU prevented the spread of MDROs
	In an Australian hospital CPE outbreak, seven patients and three carriers identified over a three-year period were linked by genome analysis
If you think the answer option would be useful in improving hospital infection control, please circle only those that apply.	National guidelines
	Guidelines by academic associations
	Activities to gain support from organization by other hospitals
	Patients for whom other hospitals conduct admission screening for MDROs
	Criteria by other hospitals for determining MDROs outbreaks
	Criteria by other hospitals for determining patients free from a MDROs transmission
	The timing and frequency of screening of patients who had contact with MDRO patients at other hospitals
	The cost-effectiveness of IC strategies
	Training effective on developing IC
	To share knowledge with other hospitals
	Closer communication with other hospitals
	Advice from IC specialists by visiting the hospitals

A response rate of 80.0% was obtained from 24 healthcare facilities (HCFs), including 18 hospitals and six AFs with beds [11]. The following topics were covered in the responses to the hospitals’ questionnaire: IC practices for sinks, IC practices to be conducted for sinks, first learned information, and useful information. Variables were all summarized using the number of hospitals. When we summarized and analyzed the data, answers marked with a check mark were quantified as 1, and answers without a check mark were quantified as 0. Multiple and single regression analyses were used to determine the relationship between IC practices to be conducted for sinks and the first learned information. The dependent variables were the intention to develop IC practices for sinks (dedicate sinks to handwashing, install splash guards, do not keep supplies on surfaces that are within 1 m of sinks, refrain from disposing substances such as medications and liquid food). The independent variables were the first-learned information (cleaning bundle effects, knowledge sharing organized by the PHC, effect of training session programs on IC). The statistical model is as follows: Y = β₀ + β₁X₁ + β₂X₂ + β_3_X_3 _+ ε; Y = dependent variable; β₀ = intercept; β₁, β₂, β_3_ = regression coefficients of each explanatory variable; X₁, X₂, X_3_ = independent variables; and ε = error term. Furthermore, a correlation analysis was carried out to determine the relationships between IC practices to be conducted for sinks and useful information. We used BellCurve for Excel (BellCurve, Tokyo) for multiple and single regression analyses and correlation analysis. We performed a Pearson correlation coefficient test for the correlation analysis.

The search parameters of questionnaires (Table 3) were as follows. We selected IC which the US guidelines described and which medical facilities in Kawaguchi City implemented as the answer options for sink IC. Additionally, we searched PubMed for literature from 2013 to June 2023 using the keywords “multidrug and resistant and sink.” The number of articles searched was 56, and we analyzed 28 using the method defined in the Preferred Reporting Items for Systematic Reviews and Meta-Analyses (PRISMA). The subjects of the analysis were literature on IC for sinks in hospitals. Following our analysis, we found that a sink with a depth of 24 cm or more prevents water from splashing, and we used this information as an answer option for IC regarding the sink.

The keywords by which the first-learned information were selected utilized PubMed as follows. Regarding the effectiveness of feedback and feedback methods, we searched PubMed for literature published between 2013 and June 2023 using the keywords “feedback and goal setting and hand hygiene,” “ward and visual and hand hygiene,” and “hand hygiene and feedback and impact.” The number of articles we searched was nine, 12, and 29, respectively, and we analyzed three, three, and 19 articles, respectively, using the PRISMA method. We analyzed articles that reported on feedback and goal setting for developing hand hygiene compliance, methods of visual feedback on hand hygiene compliance in wards, and feedback methods that were effective in developing hand hygiene compliance. Following our analysis, we identified a strategy on effective HHCPs, including feedback on hand hygiene compliance over the past seven days, feedback with illustrations, and feedback on staff’s personal hand hygiene compliance. We used the report that feedback is effective on hand hygiene compliance and how to provide effective feedback as answer options for the first-learned information.

Regarding the effectiveness of simulation-based training on developing hand hygiene compliance, we searched PubMed for literature published between 2013 and June 2023 using the keywords “novel and intervention and methicillin-resistant *Staphylococcus aureus *(MRSA) and hand hygiene.” We searched 10 articles and analyzed three using the PRISMA method. The articles included reports on the effects of practical educational methods on hand hygiene compliance. Following our analysis, we found that simulation-based training was effective in developing hand hygiene compliance, and we used this as an answer option for the first-learned information.

Regarding a high risk of infection when hospitalized in an ICU room where a patient with MDROs had been hospitalized, we searched PubMed for literature published between 2003 and June 2023 using the keywords “risk of acquiring multidrug-resistant and room.” The number of articles we searched was 19, and we analyzed three using the PRISMA method. We analyzed the reports that the hospital room environment might have infected patients with MDROs. We found that there was a high risk of infection when hospitalized in an ICU room where a patient with MDROs had been hospitalized. We used this finding as an answer option for the first-learned information.

Regarding the report that the number of infected patients who had indirect contact with MDRO-identified patients did not decrease despite enhanced IC, and that genome analysis linked 10 cases of CPE identified over a period of approximately three years, we searched PubMed for literature from 2013 to June 2023 using the keywords “whole-genome sequencing and CPE and outbreaks and infection control and reservoir.” We searched nine articles and analyzed five using the PRISMA method. We analyzed the reports that MDROs might be transmitted from the hospital environment. We found that the number of infected patients who had indirect contact with MDRO-identified patients had not decreased, and that 10 cases of CPE identified over a period of about three years were related. We used these as answer options for the first-learned information.

Regarding plasmid matched between CPE-identified patients in the same medical facility without epidemiological links, we searched PubMed for literature from 2013 to June 2023 using the keywords “patient and plasmid and transfer and hospital environment and carbapenemase-producing *Enterobacteriaceae*.” We searched 47 articles and analyzed 10 using the PRISMA method. We analyzed literature reporting the matching of plasmids of CPE between patients and the environment. Following our analysis, we found that the CPE plasmids between patients and between patients and sinks were identical. We used these as options for first-learned information, that plasmid matches between patients with CPE in the same medical facility with no epidemiological link, that genomic sequencing of isolates from patients revealed links to previous CPE outbreak strains, and that CPE plasmids from the patient were linked to those from sinks in the patient’s room.

Regarding the answer option that the single MRSA strain was widespread in the dust in the ward environment during the outbreak, we searched PubMed for literature published between 2003 and June 2023 using the keywords “infection control and environment and MRSA and dust.” We searched 10 articles and analyzed four using the PRISMA method. The literature we analyzed was the report on the identification of MRSA from the environment. Following our analysis, we found that the single MRSA strain was widespread in the dust in the ward environment during the outbreak, and we used this as an answer option for the first-learned information.

Regarding the identification of the same strain of multidrug-resistant *Acinetobacter *(MDRA) in ventilator dust as that found in the patient, we searched PubMed for literature published between 2003 and June 2023 using the keywords “*Acinetobacter baumannii* and dust.” We searched 13 articles and analyzed two using the PRISMA method. We analyzed literature reporting the presence of *Acinetobacter baumannii* in dust in hospital environments. Following our analysis, we found that the same strain of MDRA was identified in the dust from ventilators as in patients, and we used this as an answer option for the first-learned information.

Regarding the effect of cleaning bundles, we searched PubMed for literature published from 2013 to June 2023 using the keywords “environmental cleaning and effect and health associated infection and reduce and hospital and control.” We searched 74 articles and analyzed 11 using the PRISMA method. The literature we analyzed was the reports on a reduction in infectious diseases by enhancing environmental cleaning. Following our analysis, we found a reduction in infectious diseases and cost reduction by cleaning bundles, and we used this as an answer option for the first-learned information.

Regarding reports on effectiveness of enhancing EC, we searched PubMed for literature published between 2013 and June 2023 using the keywords “EC and sustaining and reduction and hospital.” We searched 38 articles and analyzed six using the PRISMA method. The articles analyzed were reports such that EC programs were not temporary and reduced identification of infection. We found that the identification of MDROs reduced by evaluating and providing feedback on environmental cleaning and by reassigning and hiring new cleaning staff. We used effectiveness of enhancing EC as an answer option for the first-learned information.

Regarding the effectiveness of training on IC, we searched PubMed for literature from 2013 to June 2023 using the keywords “training and MRSA and reduction” and “healthcare worker and transmission and multidrug resistant organisms.” The number of articles we searched was 116 and 47 and analyzed nine and two using the PRISMA method. We analyzed reports on the association between staff training and reduced MDROs identified by the hospitals and reports on staff knowledge developed by TSs. We found that a higher percentage of people provided with training sessions on MDROs was associated with a lower incidence of MRSA infections, and that people who had received training in the last three years had more knowledge about MDROs and IC. We used the effectiveness of training on IC as answer options for the first-learned information.

In Japan, the government leads networks for preventing health-associated infection (HAI), which consists of hospitals and PHCs. We thought that we should provide detailed information on knowledge sharing organized by PHC if the hospitals did not know about it. We used this as an answer option for the first-learned information.

Regarding the prevention of the spread of MDROs by enhancing contact precautions, we searched PubMed for literature published between 2013 and June 2023 using the keywords “contact precautions and MDRO and isolation.” We searched 22 articles and analyzed three using the PRISMA method. We analyzed articles on the prevention of the spread of infection by enhancing contact precautions. Following our analysis, we found that enhancing contact precautions without isolation rooms in the ICU prevented the spread of MDROs, and we used this as an answer option for the first-learned information.

We selected answer options for providing information and activities considered useful for developing IC as follows: the information that hospitals would like to know during discussions between the PHC and hospitals in Kawaguchi City, and the activities by network for preventing HAI, whose members are managers or staff of hospitals and PHC stipulated by the Japanese government.

We selected the independent variables for the following reasons. We suggested that support from hospital organizations is crucial to the development of IC programs [11]. Moreover, hospitals could develop their own IC programs, and staff could develop their own IC practices if they were provided evidence about cost-effective hospital IC [11]. Therefore, we hypothesized that the effects of cleaning bundles, which include cost reduction [13], might help staff develop IC practices for sinks and selected it as an independent variable. We selected “discussion by the hospitals on the difficulties and barriers of knowledge sharing organized by the PHC” (knowledge sharing organized by the PHC) as an independent variable, because we hypothesized that it would help the hospitals develop IC practices for sinks. The reasoning is that IC preventionists (IPs) exchange information on IC with hospitals in their region and provide the hospital management with information about IC to analyze cost-effectiveness [14]. Therefore, the hospitals might expect to be provided with information on cost-effective IC practices for sinks by the IPs after they participated in knowledge sharing organized by the PHC. Moreover, we suggested that the hospitals could develop their own IC programs when the PHC provided evidence on cost-effective information [11]. We hypothesized that hospitals could conduct IC practices for sinks after the PHC provided the effect of TSs. The reason why we selected the effect of TSs as an independent variable is that IC training was associated with higher knowledge scores regarding MDROs and a decrease in MDRO infections (Table 2, Appendix).

The role of the Japanese PHC is to ensure safety in the hospitals by guiding them to develop IC programs and practices [11-12]. The role of the hospitals is to develop their own IC programs and practices [11-12]. We carried out the TS program and the implementation of the posttraining survey based on the provisions stipulated in the Japanese Medical Care Act as follows. PHCs can inspect the hospitals’ hygiene conditions, including IC, and give instructions to develop them if they found find any deficiencies. PHCs provide information regarding medical safety, including IC. In this study, we aimed to clarify effective strategies by the PHC to develop IC practices for sinks in its jurisdiction, and we analyzed the survey results that were defined as activities by Kawaguchi City as stipulated by law and examined them based on research report recommendations and best practices. This study involved no invasive procedures (e.g., drawing blood, collecting samples, or asking traumatic questions) and did not use human subjects. Hence, the ethics committee did not have to approve this study.

## Results

**Figure 1:**
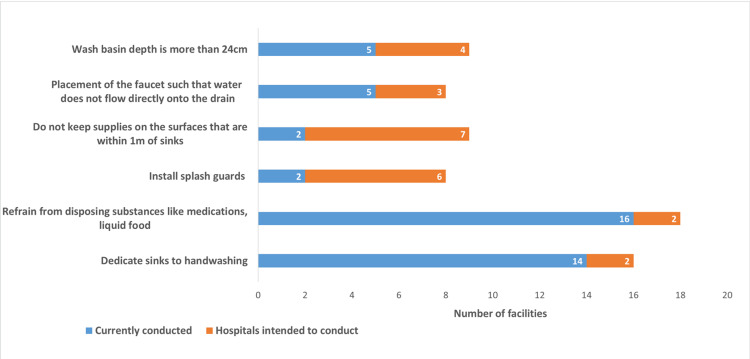
Infection control practices for sinks conducted by the hospitals and that the hospitals intended to conduct (n = 24) Data are N

Current IC practices for hospital sinks included refraining from disposing substances like medications and liquid food (refrain from disposing substances ) by 16 hospitals (66.7%), dedicating sinks for handwashing by 14 hospitals (58.3%), installing faucets that prevent water from flowing directly into the drain by five hospitals (20.8%), installing wash basins more than 24cm deep by five hospitals (20.8%), installing splash guards by two hospitals (8.3%), and keeping supplies off surfaces within 1m of sinks by two hospitals (8.3%; Figure 1). 

IC practices to be conducted for sinks included refraining from disposing substances by two hospitals (8.3%), dedicating sinks to handwashing by two hospitals (8.3%), installing splash guards by six hospitals (25.0%), and keeping supplies off nearby surfaces by seven hospitals (29.2%; Figure 1). Components of the first learned information included the effects of cleaning bundles by 10 hospitals (41.7%), knowledge sharing organized by the PHC by four hospitals (16.7%) and the effect of TSs by three hospitals (12.5%; Table 4).

**Table 4:** The first learned information from training sessions (n = 24) MRSA: methicillin-resistant *Staphylococcus aureus*; MDRA: multidrug-resistant *Acinetobacter*; EC: environmental cleaning; IC: infection control; CPE: carbapenemase-producing *Enterobacteriaceae* Data are N (%)

Description	Number of facilities	Percentage (%)
MRSA strain was widespread in the dust in the ward environment in the outbreak	12	50.0
MDRA outbreak strains were isolated from the dust of respiratory ventilators	11	45.8
Implementing the cleaning bundles resulted in preventing healthcare-associated infections and cost savings	10	41.7
Enhanced EC practices were effective for IC	9	37.5
Specific feedback was effective for hand hygiene compliance programs	8	33.3
Despite implementing enhanced IC measures, the transmission of CPE through indirect ward and hospital contact did not decrease	8	33.3
Sequencing of CPE isolates from patients revealed links to previous outbreak strains	8	33.3
Training simulations were effective for hand hygiene compliance programs	7	29.2
Feedback was effective for hand hygiene compliance programs	6	25.0
Discussion of the difficulties and barriers to developing IC in knowledge sharing organized by the public health center were helpful	4	16.7
The training sessions were effective for IC	3	12.5

**Figure 2:**
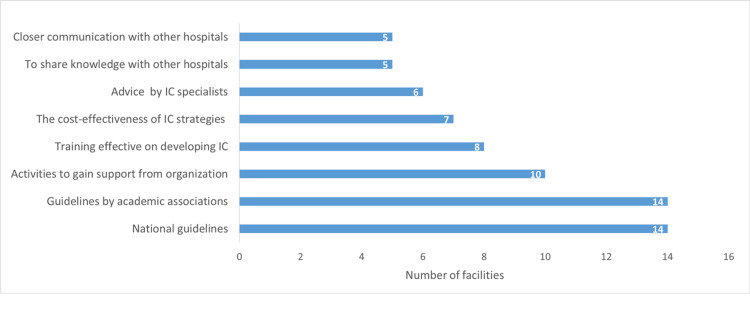
Useful information and activities for developing IC by the hospitals (n = 24) IC: infection control Data are N

Useful information included “national guidelines” identified by 14 hospitals (58.3%), “guidelines by academic associations” identified by 14 hospitals (58.3%), and “activities to gain support from organizations” (activities for organizations) identified by 10 hospitals (41.7%; Figure 2).

The first learned information, “the effect of TSs,” was significantly associated with the intention to dedicate sinks to handwashing (standard partial regression coefficient = 0.62, p = 0.008; Table 5). The following IC practices to be conducted for sinks were not associated to the first learned information: refraining from disposing substances, installing splash guards, and keeping supplies off nearby surfaces (Table 5).

**Table 5:** Association between the first learned information from training sessions and IC practices that hospitals intended to conduct regarding sinks (n = 24) IC: infection control; RC: regression coefficient; CI: confidence interval Results by multiple regression analysis and single regression analysis. Statistical significance determined as p ≤ .05

Dependent variables	Independent variables	Multiple regression analysis			Single regression analysis		
IC regarding practices that hospitals intend to conduct	The first learned information from training sessions	Standard partial RC	p-value	95% CI	Standard partial RC	p-value	95% CI
Dedicate sinks to handwashing	Cleaning bundles effects	-0.34	0.076	-0.40-0.02	-0.25	0.230	-0.38-0.10
	Knowledge sharing organized by the public health center	-0.42	0.055	-0.64-0.01	-0.13	0.530	-0.42-0.22
	Effect of training session programs on IC	0.62	0.008	0.15-0.89	0.34	0.102	-0.06-0.63
Install splash guards	Cleaning bundles effects	-0.17	0.419	-0.53-0.23	-0.10	0.650	-0.47-0.30
	Knowledge sharing organized by the public health center	0.09	0.695	-0.46-0.68	0.26	0.223	-0.20-0.80
	Effect of training session programs on IC	0.35	0.163	-0.20-1.11	0.36	0.081	-0.06-1.02
Do not keep supplies on surfaces that are within 1m of sinks	Cleaning bundles effects	-0.04	0.839	-0.46-0.37	0.02	0.943	-0.39-0.42
	Knowledge sharing organized by the public health center	0.06	0.804	-0.55-0.70	0.21	0.337	-0.28-0.78
	Effect of training session programs on IC	0.29	0.261	-0.32-1.11	0.31	0.138	-0.15-1.01
Refrain from disposing substances like medications and liquid food	Cleaning bundles effects	-0.33	0.114	-0.42-0.05	-0.25	0.230	-0.38-0.10
	Knowledge sharing organized by the public health center	0.12	0.603	-0.26-0.44	0.27	0.203	-0.12-0.52
	Effect of training session programs on IC	0.34	0.151	-0.11-0.69	0.34	0.102	-0.06-0.63

A strong correlation was found between the intention to install splash guards and the intention to keep supplies off with a correlation coefficient of 0.688 (Table 6). The correlation coefficients between the independent variables (effects of cleaning bundles, knowledge sharing organized by the PHC, and effects of TSs) were 0.076, 0.192, and 0.507, respectively (Table 6), and we deemed multicollinearity negligible. No correlation existed between the intention to dedicate sinks and install splash guards and activities for organization, with correlation coefficients of 0.051 and 0.098, respectively (Table 7).

**Table 6:** Correlation between IC practices that hospitals intended to conduct regarding sinks and the first learned information (n = 24) IC: infection control. *Pearson rank correlation coefficient Statistical significance determined as p ≤ 0.05

Variable 1	Variable 2	* Pearson rank correlation coefficient (between variables 1 and 2)	p-value
IC practices that hospitals intended to conduct regarding sinks			
Dedicate sinks to handwashing	Install splash guards	0.522	0.009
Dedicate sinks to handwashing	Do not keep supplies on surfaces within 1 m of sinks	0.470	0.021
Dedicate sinks to handwashing	Refrain from disposing of substances like medications and liquid food	0.455	0.026
Install splash guards	Do not keep supplies on surfaces within 1 m of sinks	0.688	0.000
Install splash guards	Refrain from disposing of substances like medications and liquid food	0.522	0.009
Do not keep supplies on the surfaces that are within 1m of sinks	Refrain from disposing of substances like medications, liquid food	0.470	0.021
The first learned information from training sessions			
Implementing the cleaning bundles resulted in preventing healthcare-associated infections and increased cost savings	Discussion by the hospitals on the difficulties and barriers of knowledge sharing organized by the public health center	0.076	0.726
Implementing the cleaning bundles resulted in preventing healthcare-associated infections and increased cost savings	The training session programs were effective for infection control	0.192	0.370
Discussion by the hospitals on the difficulties and barriers of knowledge sharing organized by the public health center	The training session programs were effective for infection control	0.507	0.011

**Table 7:** Correlation between IC practices that hospitals intended to conduct regarding sinks and useful information and activities on developing IC by the hospitals (n = 24) IC: infection control. *Pearson rank correlation coefficient Statistical significance determined  as  p ≤ 0.05

Variable 1 IC practices that hospitals intended to conduct regarding sinks	Variable 2 Useful information and activities on developing IC	Pearson correlation coefficient (between variable 1 and 2〕	p-value
Dedicate sinks to handwashing.	Activities to gain support from organizations by other hospitals	0.051	0.813
	Evaluation of the cost-effectiveness of infection control strategies by other hospitals	0.138	0.520
	Effective training on developing IC	-0.213	0.317
	Advice provided and hospitals visited by infection control specialists	0.174	0.416
Install splash guards.	Activities to gain support from organizations by other hospitals	0.098	0.650
	Evaluation of the cost-effectiveness of infection control strategies by other hospitals	0.053	0.806
	Effective training on developing IC	0.204	0.339
	To be provided advice and visiting the hospitals by infection control specialists	0.333	0.111
Do not keep supplies on the surfaces that are within 1m of sinks	Activities to gain support from organizations by other hospitals	0.201	0.345
	Evaluation of the cost-effectiveness of infection control strategies by other hospitals	0.193	0.366
	Training effective on developing IC	0.519	0.009
	Advice provided and hospitals visited by infection control specialists	0.265	0.211
Refrain from disposing substances like medications, liquid food	Activities to gain support from organizations by other hospitals	0.357	0.087
	Evaluation of the cost-effectiveness of infection control strategies by other hospitals	0.138	0.520
	Effective training on developing IC	0.107	0.620
	Advice provided and hospitals visited by infection control specialists	0.522	0.009

## Discussion

Strategies for developing IC practices for sinks

Microorganisms that proliferate in the sink tend to form biofilms, and once they form, they are difficult to destroy from the outside [15]. Moreover, drug-resistant genes have been identified in microorganisms in the sinks [15]. The WHO and Japanese government do not specify dedicating sinks for handwashing in their guidelines, although this strategy is specified in the Society for Healthcare Epidemiology of America (SHEA) guidelines in the United States [7]. If only water and soap are disposed of in the sink, MDROs would rarely be identified [16]. Nevertheless, once nutrients are discarded in the sinks, MDROs are often identified immediately [16]. We suggest that more MDROs might be hidden in the hospital sinks than are identified in the hospitals in Kawaguchi City, where 14 (58.3%) hospitals dedicate sinks to handwashing (Figure 1). We suggest that public health authorities including the PHC (public health authorities) and IC specialists who provide the hospitals with advice on IC (IC specialists) might need to guide the hospitals to dedicate sinks to handwashing.

Moreover, in its guidelines, the provincial government of Canada recommends that medical equipment should not be washed in handwashing sinks [17]. In Japan, medical equipment is cleaned in a separate washing sink from the handwashing sink [18]. Meanwhile, there is a report that medical items are cleaned in hand washing sinks as a common practice in somewards [19]. We suggest that the public health authorities and IC specialists might need to inform the hospitals that MDROs are often identified immediately after nutrients are disposed of in the sinks [16] and guide them not to dump liquids that include nutrients and not to wash medical items in handwashing sinks.

Staff members who were not working or had not previously worked in IC roles often disposed of liquid waste in handwashing sinks without awareness that the sinks could be a source of infection [20]. In our TSs, we indicated that hospitals with a high percentage of staff who received training on MDROs have a lower incidence of MRSA infections (Table 2).Additionally, we indicated that enhanced EC including staff training has developed EC and reduced HAIs (Table 2) [13]. Moreover, the training developed hand hygiene compliance and reduced catheter-related bloodstream infections [21]. Therefore, we suggest that hospitals recognize the effectiveness of training sessions on changing staff behavior, such as environmental cleaning and hand hygiene. Moreover, we suggest that hospitals can expect their staff to refrain from disposing of substances such as medications and liquid food if they provide training sessions. Therefore, we suggest that there was an association between “dedicate sinks to handwashing” and “the effect of TSs on IC (Table 5).” Public health authorities and IC specialists might need to provide the hospitals with information on the effect of TSs on IC to guide the hospitals to dedicate sinks to handwashing.

Hospitals must prepare a container for disposing and storing waste liquids temporarily and should gain the cooperation of the patients to address the problem of disposing substances in hospital sinks. Moreover, we suggest that a space to store liquids that were originally emptied into the sink might be needed in staff areas. Therefore, we suggest that the first learned information, which included “effects of cleaning bundles,” “knowledge sharing organized by the PHC,” and “the effect of TSs on IC,” were not associated with refraining from disposing of substances to be conducted (Table 5).

The WHO and Japanese government do not specify installing splash guards in their guidelines, unlike the SHEA guidelines in the United States [7]. It has been reported that many bloodstream infections caused by MDROs have been identified in hospitals where water splashes have been observed around the sinks [22]. Moreover, direct water flow into the drain could disperse the pathogen [15]. To address this issue, it has been reported that installing a drain cover can contribute to preventing pathogens from being scattered around the sink even in the absence of splash guards [23]. Likewise, installing a faucet that keeps the water from flowing directly into the drain in addition to installing splash guards reduced the amount of water splashing from the sinks [10]. However, a wash basin that is deeper than 24 cm greatly reduced the amount of water splashing around the basin, preventing the spread of pathogens without the installation of splash guards [24]. In Kawaguchi City, only two hospitals (8.3%) had installed splash guards, five (20.8%) installed a faucet that keeps the water from flowing directly into the drain, and five (20.8%) installed sinks deeper than 24 cm. The percentage of hospitals that conducted these IC practices was extremely low (Figure 1). Therefore, we suggest that public health authorities and IC specialists need to guide the hospitals to install splash guards, drain covers [23], or faucets that keep the water from flowing directly into the drain, thereby preventing the spread of pathogens without replacing sinks. Nevertheless, we suggest that the ward structure around the basin (i.e., the location of the handwashing sinks in the patients’ rooms) interferes with the installation of splash guards, even if the hospitals intend to install them. Therefore, we suggest that the pieces of first learned information “effects of the cleaning bundle,” “knowledge sharing organized by the PHC,” and “the effect of TSs on IC” were not associated with the intention to install splash guards (Table 5).

The WHO and Japanese government do not specify keeping supplies off countertops or mobile surfaces that are within 1 m of sinks in their guidelines, although this practice is specified in the SHEA guidelines [7]. Actions occurring around the sink in the rooms of the ICU patients have been observed [19]. Most of these behaviors involved both staff and patients moving back and forth between the sinks and the patients’ beds, indicating that the sinks might be a source of infection [19]. Furthermore, in sink-related outbreaks, items around handwashing sinks were associated with the spread of infection [15] and splashes of water more than 1 m from the washing sink have been observed [25]. In Kawaguchi City, only two hospitals kept supplies away from surfaces within 1 m of sinks (Figure 1). There is a report that patient care items were left around the sinks before the staff flushed water and medicine down the drains in the ICUs patients’ rooms [19]. Rooms without enough flat space for patients to leave their belongings often had patient care items left around the sink [19]. We suggest that the lack of usable space to keep patient care items in hospitals contributes to placing supplies on surfaces within 1 m of sinks in the hospital wards, even though the hospitals prefer not to do so. Therefore, we suggest that the first learned information “effects of the cleaning bundle,” “knowledge sharing organized by the PHC,” and “the effect of TSs on IC” were not associated with keeping supplies off surfaces within 1 m of sinks to be conducted (Table 5). We propose that public health authorities and IC specialists need to guide the hospitals to ensure spaces where water will not splash on supplies. Additionally, public health authorities and IC specialists should conduct a survey to determine the reason why the hospitals place items within 1 m of the sinks.

Organizational support and organizational barriers

No correlation existed between HHCPs and screenings of MDROs upon hospital admission that the hospitals intended to implement and organizational activities [11]. Information on cost-effectiveness was used to encourage the hospitals to develop IC [11]. Additionally, the hospital management often decides on installing sinks in wards [26]. Therefore, we suggest that no correlation existed between the intention to dedicate sinks to handwashing and install splash guards on the one hand and activities to obtain support from the hospital organization the other (Table 7). Information on cost-effectiveness must be provided to gain support from staff and hospital leadership [11]. Therefore, we suggest that providing information on cost reductions might be effective for encouraging the hospitals to conduct the relevant practices. However, to our knowledge, there have been no reports on cost reductions achieved by enhancing IC practices for sinks. We propose that research on the cost-effectiveness of enhanced IC practices for sinks should proceed in the future.

TS programs to encourage the development of IC practices for hospital sinks

Providing evidence that TS programs were effective in developing IC might incline more hospitals to dedicate sinks for handwashing (Table 5). We suggest that the hospitals expect their staffs to avoid using the sinks for activities other than handwashing and that they would dedicate sinks to handwashing in the future after learning of the effectiveness of the TSs. We propose that public health authorities and IC specialists need to provide information that is effective for developing IC practices by medical staff to encourage hospitals to dedicate sinks to handwashing. Our 2023 study indicated that the first piece of learned information, “the effects of cleaning bundles which include staff training on environmental cleaning” [13], could develop HHCPs [11]. Staff support is crucial not only for developing HHCPs but also for dedicating sinks to handwashing [20]. We suggest that public health authorities and IC specialists should provide the hospitals with information about the effects of TSs and education, addressing the development of IC practices that cannot be strengthened without staff support.

Strengths and limitations of this study

In this study, we found that hospitals were willing to dedicate sinks to handwashing, a practice that cannot be implemented without staff support, when the PHC provided evidence of the effect of TSs on IC. We indicated that the public health authorities and IC specialists should provide the hospitals with information that MDROs are more likely to be identified if nutrients are flushed down the drain [16] and should guide them not only to refrain from disposing of substances like medications and liquid food but also to desist from cleaning medical items in handwashing sinks. Moreover, we indicated that public health authorities and IC specialists should inform hospitals that installing splash guards can help prevent pathogens from being scattered. We indicated that the public health authorities and IC specialists should guide hospitals to ensure available space in the wards to place items where they will not get splashed and should conduct a survey to discover why hospitals place items within 1 m of sinks. We suggested that public health authorities and IC specialists should provide the hospitals with information on the positive effects of TSs and education, addressing the development of IC practices that cannot be strengthened without staff support. Furthermore, we informed the hospitals that after we compiled the survey results, we would edit them to prevent identification of the names of the hospitals and those who had filled out the responses before publication. Additionally, we provided TS logistics and specific questionnaires so that other PHCs could replicate our program. To examine the TSs effective for developing IC for sinks, we used multiple regression analysis to analyze the relationship between the first-learned information from TSs and the intention to develop IC for sinks. To our knowledge, except for our 2022 and 2023 studies, no researchers have used this method to analyze effective TS programs in developing IC. The survey response rate in this study was relatively high (80.0%). This enabled us to collect information on IC practices related to hospital sinks in hospitals throughout the city. We anticipate that specific, effective TSs on IC practices for sinks will not only help hospitals in other districts develop IC practices but will also strengthen the PHC’s support of these methods.

This study has several limitations. First, the information collected only covered 24 hospitals in Kawaguchi City, resulting in a small sample size and may not fully represent IC practices in other regions. Second, TS programs associated to and not associated to IC practices for sinks might not be applicable in other districts where the guidelines for IC practices for sinks are available. Third, we did not examine current IC practices for hospital sinks to determine the effect of TS programs. There is no guarantee that the intention to develop IC for the hospital sinks we examined will translate into development of staff’s actual IC behaviors. We suggest conducting direct observation and follow-up surveys to determine whether the hospitals’ intention is to develop IC into IC practices. Additionally, we suggest that the hospital mistakenly did not mark the answer option that it should have answered. Moreover, our suggestions and proposal listed below lack evidence. The suggestion on the reason why “the effect of the cleaning bundle,” “knowledge sharing organized by the PHC,” and “effect of TSs on IC” were not associated with refraining from disposing of liquids in the sink, installing splash guards, and keeping supplies off surfaces within 1 m of sinks. The proposal on conducting a study on cost-effectiveness by enhancing IC practices for hospital sinks.

However, we expect that the program implemented in this study, which aimed to provide the hospitals with effective TSs for developing IC practices for sinks, will serve as a model for other jurisdictions. Additional surveys and evaluations in different settings will help determine the contributions made by the strategies used in this study to the development of IC practices for sinks.

## Conclusions

This study and its effects should be examined in other districts. Moreover, direct observation and follow-up surveys should be conducted in the future to determine whether staff have translated the hospitals’ intention to develop IC into IC practices. However, we can conclude that training programs can help hospitals in developing IC practices that cannot be strengthened without staff support. In light of these finding, we suggest that the PHC continue to provide hospitals under its jurisdiction with effective TSs on IC practices for sinks and guide hospitals to develop their own IC practices.

## Appendices

**Table 8:** Evidence and recommendation on training session programs on IC and prevention of MDROs by Kawaguchi City Public Health Center IC: infection control; MDROs: multidrug-resistant organisms; MRSA: methicillin-resistant *Staphylococcus aureus*; NICU: neonatal intensive care unit; EC: environmental cleaning; ICU: intensive care unit; CPE: carbapenemase-producing *Enterobacteriaceae*; MDRA: multidrug-resistant *Acinetobacter*; CRE: carbapenem-resistant *Enterobacteriaceae*; CDC: Centers for Disease Control and Prevention

Training session topics	Evidence and recommendation on training session programs
Hand hygiene compliance programs	Hand hygiene compliance before patient contact in Japanese hospitals was 19.1% and might need further improvement
	Performance feedback may increase adherence to hand hygiene practices and reduce infection and colonization rates
	A ward-level hand hygiene compliance program can be successful in improving hand hygiene rates
	Hospitals that provided feedback with visually appealing illustrations improved their hand hygiene compliance
	Feedback on the mean compliance rate for the past seven days should be provided
	Individual compliance rates were publicly posted in designated areas. We recommend that you provide the staff with feedback for a ward or department, and that such feedback enables the staff to see the rate of hand hygiene compliance
	Simulation training sessions were effective for hand hygiene compliance programs. During an MRSA outbreak, simulation-based training session programs were conducted in the NICU, presenting actual situations in which IC practices were necessary (e.g., an infusion pump alarm sounded). Hand hygiene compliance rates remained improved for approximately six months after the training session programs ended
	Each ward should be responsible for setting its own goal for the hand hygiene rate. In hospitals where hand hygiene compliance rates had improved, their staff set goals over the next four weeks
	Hand hygiene champions, doctors, and nurses should lead hand hygiene compliance programs. It is important that staff be involved in activities to develop hand hygiene compliance
	Targets of 10–20% hand hygiene improvement were set
	It is important to set reasonable goals for hand hygiene compliance that are slightly higher than the current level. Once the goals are achieved, we recommend maintain the frequency of hand hygiene
Evidence on enhanced EC	Admission to an ICU room previously occupied by a patient with MDROs was a risk factor for acquisition of the bacteria by subsequent room occupants
	Despite implementing enhanced IC, including contact precautions, the transmission of CPE through indirect ward and hospital contact did not decrease. The enhanced IC programs to prevent the spread of infection are as follows: endoscope infection surveillance, terminal room disinfection (using sodium hypochlorite and hydrogen peroxide vapor), private room management and cohort management for CPE carriers, antibiotic stewardship programs, hand hygiene compliance programs, contact precaution (gowns, aprons, gloves), screening of contacts identified by epidemiological investigations, screening of patients (when high-risk patients are admitted, when transferring to or from high-risk wards)
	Genomic sequencing of isolates from patients revealed links to previous CPE outbreak strains from two years prior. Genomic analysis revealed that two patients who had been admitted to the facilities organized by the same healthcare systems and were not epidemiologically linked had almost identical strains and the same plasmid genes
	Implementing the cleaning bundle resulted in a decrease in MDROs and cost savings. The incidence of hospital-acquired infections was reduced after education for cleaning staff by nurses, evaluation and feedback of environmental cleaning, and the reassignment and hiring of new cleaning staff
Evidence for dust removal and EC that hospitals should conduct	We recommend that the hospitals remove dust in relevant areas by mopping, in reference to IC policies by university hospitals
	During the outbreak, the single MRSA strain was widespread in the dust in the ward environment. There is a report that recommends removing dust because the medical staff might transmit MRSA
	The MDRA outbreak strains were isolated from the dust of respiratory ventilators and continuous veno-venous hemofiltration. During the outbreak, MRSA was found on furniture, floors, medical equipment, beds, surfaces, door handles, ventilation ducts, heating appliances (36.4%, 16 of 44 samples), and nurse call buttons
Evidence for cleaning sinks and drains and EC that hospitals should conduct	Do not keep medications or patient care supplies on surfaces that are within 1 m of sinks
	A splash barrier should be used
	The sink has a basin depth of at least 240 mm
	Covers should be put on sink drains
	CPE plasmids from the patient were linked to those from sinks in the patient’s room. Drain cover has also been effective in preventing the spread of gram-negative bacteria. By removing sinks, implementing hand hygiene by wipes and alcohol-based gel, and using disposable medical equipment, the prevalence of gram-negative bacilli in the ICU decreased from 26.3% to 21.6%
Evidence for monitoring of and feedback about EC	The possibility of transmission through inanimate objects could not be ruled out in the MDRO outbreak
	Monitoring of and feedback from EC is recommended.
	Enhanced EC practices, including monitoring and feedback, and hiring new personnel can reduce MDROs infection rates.
	The incidence of hospital-acquired infections was reduced by providing feedback to staff engaged in environmental cleaning and management supervisors, by nurses evaluating whether any stains remained with fluorescent marker, and by providing feedback to the head of the cleaning department. Environmental cleaning leadership inspected the environmental cleaning by the staff and provided them with feedback. The collected data was utilized for the education of the cleaning staff. As a result, the thoroughness of environmental cleaning in medical facilities proceeded.
Knowledge sharing organized by the public health center	The hospitals discussed the difficulties and barriers to developing IC in knowledge sharing organized by the public health center.
Evidence on holding training sessions	A higher percentage of staff with IC training was associated with a decrease in MRSA infections. IC training within the last three years was associated with higher knowledge scores regarding MDROs. We recommend that you conduct training sessions in reference to IC indicated by our training session program and the results of post-training questionnaires as well as the contents of your hospital-acquired infection control policy for MDROs.
Evidence on preparing outbreaks	In a CPE outbreak at an Australian hospital, seven patients and three carriers identified over approximately three years were linked by genomic analysis. Therefore, in hospitals where CRE is sporadically identified, an outbreak may actually be occurring. Even if one case of a carrier is identified, there may be, or may have been, unidentified MDRO-positive patients in the surrounding area. If the hospitals identify even one case of MDROs, they must implement measures to prevent the spread of infection and prevent an outbreak.
The necessity and evidence for implementing measures to control MDROs throughout the jurisdiction	In New York State, local government officials visit medical institutions without an outbreak where CRE is frequently identified and exchange opinions on the implementation of IC and factors that interfere with it. When the CRE outbreak occurred in Florida, the state government cooperated with the CDC to support hospitals throughout the region in addition to those with identified patients, and the outbreak was eventually contained. Support from medical institutions is crucial in combatting and decreasing MDROs, even if there are no outbreaks in the region.
